# Structural features, interaction with the gut microbiota and anti-tumor activity of oligosaccharides

**DOI:** 10.1039/d0ra00344a

**Published:** 2020-04-24

**Authors:** Yulin Wu, Yinning Chen, Yingfang Lu, Huili Hao, Jun Liu, Riming Huang

**Affiliations:** Guangdong Provincial Key Laboratory of Food Quality and Safety, College of Food Science, South China Agricultural University Guangzhou 510642 China DanielWu@stu.scau.edu.cn luyingfang@stu.scau.edu.cn hhlhaohuili@stu.scau.edu.cn huangriming@scau.edu.cn +86 20 8528 3448 +86 7592388240; Guangdong Polytechnic College 526100 Zhaoqing China Yinning_Chen@163.com; Laboratory of Pathogenic Biology, Guangdong Medical University Zhanjiang 524023 China lj2388240@gdmu.edu.cn

## Abstract

Some oligosaccharides are regarded as biological constituents with benefits to human health in an indirect way. They enter the intestinal tract to be fermented by the gut microbiota, causing changes in the abundance and composition of the gut microbiota and producing fermentation products such as short-chain fatty acids (SCFAs). In this review, the structural features and biological activities of eight common natural oligosaccharides were summarized, including human milk oligosaccharides (HMOS), xylo-oligosaccharides (XOS), arabinoxylo-oligosaccharides (AXOS), isomaltooligosaccharides (IMOS), chitin oligosaccharides (NACOS), mannan-oligosaccharides (MOS), galacto-oligosaccharides (GOS) and fructo-oligosaccharides (FOS). Furthermore, XOS were selected to explain the anti-tumor mechanism mediated by gut microbiota. The review aims to reveal primary structural features of natural functional oligosaccharides related to the biological activities and also provide an explanation of the anti-tumor activity of functional oligosaccharides mediated by the gut microbiota.

## Abstract 2

Some oligosaccharides are regarded as biological constituents with benefits to human health in an indirect way.

## Graphical abstract

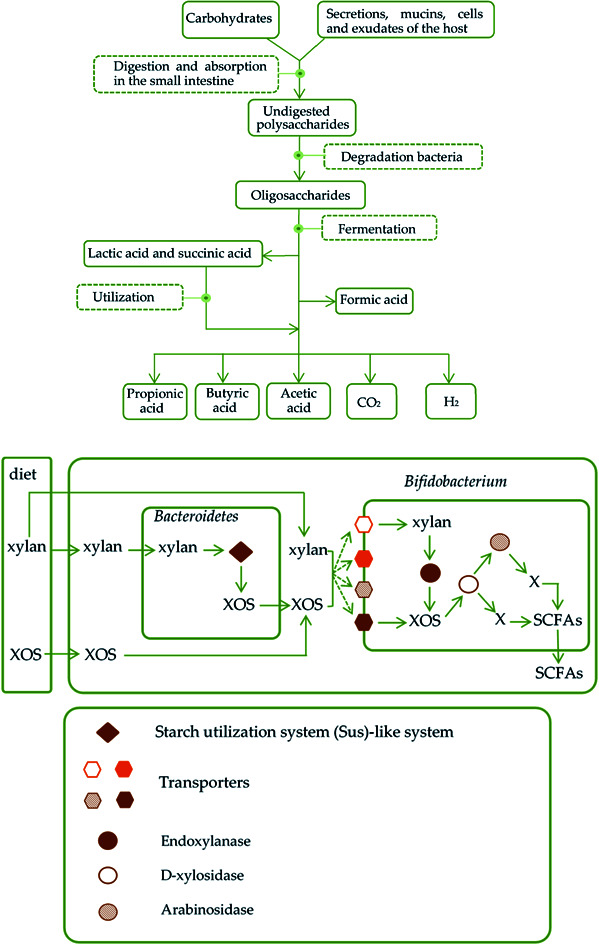

## 1 Introduction

Functional food ingredients, especially functional oligosaccharides, have been increasingly developed for human health.^1^ Oligosaccharides are low-polymerized carbohydrates composed of 2 to 10 monosaccharides linked by glycosidic bonds. Instead of being directly digested and absorbed, they enter the large intestine and are utilized by bacteria due to the absence of an enzyme system that hydrolyzes these oligosaccharides in the gastrointestinal tract of the human body.^2^ Some oligosaccharides are prebiotics, usually used as proliferation factors by beneficial bacteria.^3^ It has been shown that functional oligosaccharides contribute to various biological activities, including regulation of blood sugar and blood lipids, prevention of intestinal diseases, prevention of obesity, and anti-tumor activity.^4,5^ Some functional oligosaccharides, such as lignin-carbohydrate complexes (LCCs), also play a role in antioxidant activity.^6^ Recently, researchers have paid more attention to some oligosaccharides for their contribution to biological functions, especially the anti-tumor activity.^6^ Unfortunately, although oligosaccharides play a significant role in anti-tumor activity mediated by gut microbiota, the mechanism has not been reported comprehensively.

In recent years, the gut microbiota has attracted extensive attention thanks to their unique effects on human biological and pathological activities.^7^ Diverse microbial communities in the human intestinal tract are known as gut microbiota, and its number is estimated to be more than 1014 orders of magnitude.^8^ Gut microbiota plays a significant role in various biological processes of human body, such as digestion,^9^ synthesis,^10^ host defense^11^ and obesity.^12^ Most of the oligosaccharides, including arabinoxylo-oligosaccharides (AXOS),^13^ xylo-oligosaccharides (XOS),^14^ isomaltooligosaccharides (IMOS),^15^ human milk oligosaccharides (HMOS)^16^ and chitin oligosaccharides (NACOS)^17^*etc.*, can't be directly digested by human. But they can be degraded and utilized by gut microbiota. And some oligosaccharides can selectively proliferate beneficial bacteria and inhibit the growth and reproduction of harmful bacteria, so as to improve the abundance and composition of gut microbiota.^18,19^ Meanwhile, functional oligosaccharides can also be fermented by gut microbiota to produce products with biological activities such as SCFAs. Bering^20^ found that HMOS can serve as prebiotics and immunomodulators for preterm infants, stimulating gut adaptation and reducing the incidence of necrotizing enterocolitis (NEC). Kjolbaek^13^ found that the addition of AXOS can play a role in resisting obesity and metabolic syndrome of the host-mediated by the gut microbiota. Although many studies have proved that functional oligosaccharides can exert corresponding biological activities through the influence on gut microbiota, these studies are relatively scattered and independent. Therefore, it is of great significance to review the relationship between functional oligosaccharides and gut microbiota as well as the mechanism of their biological activities mediated by gut microbiota.

With the rapid social development, people's living standard continues to improve. And at the same time, the incidence of chronic diseases and metabolic syndrome (such as obesity, hyperglycemia, hyperlipidemia, hypertension and tumor, *etc.*) increases sharply. What's more, currently there is no effective control of a high incidence of chronic diseases and metabolic syndrome at home and abroad. With the advent of the era of precision medical and big data, people realize that the human micro-ecosystem, especially the gut micro-ecosystem plays a more and more significant role in health and disease, and even has relations with chronic diseases and metabolic syndrome in certain degree. At present, relevant scientific research teams worldwide have made brilliant achievements and outstanding contributions in the research and development field of preventing and treating chronic diseases and alleviating sub-health. But these studies are relatively isolated and short of systematic research on the molecular mechanism of oligosaccharides regulating tumor metabolism by gut microbiota. Therefore, it is of great importance to understand the anti-tumor process of oligosaccharides mediated by gut microbiota, which provides ideas for exploring new types of treatments of tumor diseases.

In this paper, the biological activities and structural characteristics of natural functional oligosaccharides were reviewed. The effects of XOS on gut microbiota were investigated as an example, and the process of anti-tumor activity mediated by gut microbiota was reviewed.

## 2 Biological activities and molecular structures of oligosaccharides

Oligosaccharides are composed of different monosaccharides linked by glycosidic bonds in various ways.^21–23^ The most common natural oligosaccharides involve HMOS, XOS, AXOS, IMOS, NACOS, mannan-oligosaccharides (MOS), galacto-oligosaccharides (GOS) and fructo-oligosaccharides (FOS).

Human milk plays a vital role in the brain development of infants.^24^ HMOS are a group of oligosaccharides biosynthesized from lactose in the mammary gland. In fact, HMOS are the third-most abundant solid component in maternal milk after lactose and lipids, and are thus considered to be key components.^25^ HMOS have inhibitory effects on the adhesion of microbiota to the intestinal mucosa, the growth of pathogens through the production of bacteriocins and organic acids, and the expression of genes that are involved in inflammation.^25^ As a kind of prebiotics, HMOS selectively promote the growth of symbiotic gut bacteria over pathogens to protect the host. They can also prevent the host from infection by playing the roles of decoy receptors that bind pathogens to inhibit cellular adhesion.^26^ The studies of HMOS cause great interest of researchers, especially in the research of infant formula milk powder instead of breast milk because of the unique biological functions of HMOS and their significant roles in the growth of infants.

XOS, the hydrolysis products of xylan, are a new type of functional oligosaccharides. Natural XOS, generally in a small account, are found mainly in plants, such as olive,^27^ sugarcane,^28^ hardwood^29^ and poplar.^30^ XOS with the polymerization degree 2–4 (ref. 31) play roles in anti-tumor activity in two aspects. Firstly, XOS have a highly selective proliferation effect on Bifidobacteria in the intestinal tract.^22,32^*Bifidobacterium* is an important probiotic in the intestinal tract, with the ability to inhibit the growth of harmful bacteria and reduce the production of endogenous tumor-causing substances. Bifidobacteria can also exert anti-tumor effects by increasing the number of immune cells and enhancing the activity of immune cells. Secondly, XOS contribute to the production of SCFAs, which have significant anti-tumor effects. And the generation of SCFAs reduces the pH of the intestinal tract and inhibits the growth of pathogenic bacteria and harmful bacteria, thus reducing the incidence of tumors.^33^ Meanwhile, XOS also play significant roles in regulating blood sugar, lowering blood lipids and serum and preventing intestinal diseases.^34,35^

AXOS are a kind of enzymatic hydrolysis product of arabinoxylan (AX). In absence of exogenous xylan degradation enzyme, AX can be degraded to AXOS by degradation enzyme such as xylanase. The degradation enzyme can be secreted by the bacteria such as Bacteroidetes in the intestinal tract.^36^ AXOS are transported into bacteria by corresponding transport carrier and further degraded by glycoside hydrolases to produce monosaccharide such as xylose and arabinose.^37^*L. acidophilus*, *L. brevis*, and *Bifidobacterium* species, for instance, produced enzymes to perform these processes.^38^ Besides, the degree of substitution of the arabinosyl side chain of AX can affect the fermentation of AX and AXOS. And the side chain groups of ferulic acid also play a role in the fermentation of AX and AXOS. The more ferulic groups there are, the harder it is for AX and AXOS to be degraded.^39^ AXOS can be used by gut microbiota to produce SCFAs. According to related researches, AXOS with a molecular weight less than 400 Da has better activity.^36^ Butyric acid can be used as an energy source of colon cells to stimulate the growth of epithelial cells of the colon^40^ and inhibit the growth of colon tumor cells.^41^ The acetic acid and propionic acid produced by fermentation are absorbed and participate in the lipid metabolism and sugar metabolism of the body respectively at the same time.^42^

IMOs found in starch generally exist as components of amylopectin or polysaccharides and are rarely found naturally as free state except in some fermented food (such as soybean paste, sake and soy sauce) as well as honey.^35^ IMOs (average molecular weight 900 Da) can be degraded by gut microbiota to improve the intestinal environment,^23^ for example, selectively promoting the proliferation of Bifidobacteria,^43^ and maintaining the balance of intestinal microbiota.^44^ Meanwhile, IMOs can also prevent the systemic and tissue inflammation, glucose intolerance, systemic obesity and other symptoms caused by hand, foot and mouth disease (HFMD) because of the production of fermentation products such as SCFAs and organic acids.

Chitin, a homopolysaccharide with the structure of *N*-acetylglucosamine polymerized by beta binding, is presented in the exoskeleton of crab and shrimp.^45,46^ NACOS can be produced *via* chitinase digestion. NACOS with the polymerization degree 2–6 have shown therapeutic effects on multiple diseases such as cancer and gastritis.^47^ NACOS also play essential roles in protection of nutrient excess-related metabolic disorders. For example, NACOS can ameliorate the metabolic syndrome caused by a high-fat diet and inhibit mRNA expression of the protein regulators related to lipogenesis as well as gluconeogenesis.^47^ NACOS can be further deacetylated into chitosan oligosaccharides (COS).^48^ COS with a low molecular weight (<1000 Da) were reported to markedly inhibit glucose uptake by suppressing the activities of pancreatic α-amylase and small intestinal α-glucosidase.^49,50^ Additionally, COS increase insulin secretion by promoting the antioxidant capacity of the pancreas,^51,52^ and exert anti-diabetic effects in rats injected streptozocin.^53^ COS possess significant biological functions with low subacute toxicity and no adverse impact at large dosage,^54^ yet the investigations on COS have rarely been done.

MOS are obtained from glucomannan of konjac through chemical or biological degradation. Studies have shown that MOS (composed of di-, tri-, tetra-, penta- and hexasaccharides, molar ratio of d-mannose to d-glucose 1.6 : 1) can attenuate metabolic syndrome induced by a high-fat diet by lowering body weight gain, lowering serum lipids and reducing insulin resistance.^55^ MOS can also modulate the overall structure of the gut microbiota, for example promoting the growth of *Lactobacillus* and *Bifidobacterium* in the cecum.^56^ Meanwhile, the composite treatment of metformin and MOS (average molecular weight *ca.* 1000 Da, mole ratio of glucose to mannose 1 : 1.2, degree of polymerization 2–6) have synergistic effects on ameliorating insulin resistance and glucose tolerance, also on repairing islet and hepatic histology.^57^

GOS are natural oligosaccharides that can be found in breast milk but rarely in animal milk. As a kind of functional oligosaccharides, GOS (comprising two to five residues of galactose terminating with an N-terminal glucose) can regulate gut microbiota to maintain intestinal health.^32^ Meanwhile, GOS are helpful to maintain healthy skin by decreasing phenols.^58^

FOS (composed of linear chains of 2 to 60 fructose units, linked by β-(2–1) bonds) are generally regarded as a type of prebiotic, inhibiting pathogens by competing with receptor sites on the gut wall and thus reducing the potential risk of infection.^59^ FOS may also prevent infection by competing effectively for nutrients with pathogens. Prebiotics manipulate the intestinal microbial environment and subsequently prevent the occurrence of infectious bowel disease.^59,60^ FOS selectively stimulate the growth of Bifidobacteria and *Lactobacilli*. However, it has shown that FOS are not only the specific substrates for these target species but can also be utilized by other bacteria such as *Streptococcus*, *Escherichia*, and *Clostridium*.^61^

It has been indicated that oligosaccharides are formed by various monosaccharides and are linked by different glycosidic bonds, and even the same kind of oligosaccharides can be formed by diverse monosaccharides in multiple ways. For example, FOS are classified into two types according to their structures. One is a linear heterooligosaccharide formed by 2–4 fructosyl through the binding of β-(2–1) glycosidic bonds on sucrose molecules. The other, formed by fructose, is mainly obtained from chicory *via* hydrolyzing by inulinase and purifying.^62^ Oligosaccharides with different modification methods can contribute to different biological functions. The structural features of eight oligosaccharides mentioned are summarized to provide foundations for studying the relationship between the structural features of oligosaccharides and their biological activities (Table 1).

**Table 1:** Structural feature of eight oligosaccharides

Number	Names	Monosaccharide composition	Sources	Modification methods	Glycosidic bonds	References
1	HMOS	d-Glucose, d-galactose, *N*-acetylglucosamine, l-fucose, *N*-acetyl-neuraminic acid	Human milk	Fucosylation, sialylation, high mannosylation		21
2	XOS	d-Xylose	Vegetable hemicellulose		β-(1–4)	22
3	AXOS	Xylose, arabinose	Enzymatic hydrolysis products of arabinoxylan (AX)	Feruloylation		22
4	IMOS	Isomaltose, panose, isomaltotriose	Cranberries, starch		α-(1–6)	23
5	NACOS	Chitosan oligosaccharides	Chitin	Acetylation	β-(1–4)	47
6	MOS	d-Mannose, d-glucose	Konjac		β-(1–3), β-(1–4)	63
7	GOS	Glucose, galactose	Milk		β-(1–3), β-(1–4), β-(1–6), α-(1–6)	64
8	FOS	Fructose, sucrose	Sucrose, chicory		β-(2–1)	65

## 3 Interaction between XOS and gut microbiota

Functional oligosaccharides can affect the abundance and composition of gut microbiota, so that they can selectively proliferate probiotics and produce metabolites with biological activity such as SCFAs.^66^ Functional oligosaccharides can promote the production and absorption of certain essential micronutrients and short chain fatty acids (SCFAs; primarily butyric acid, propionic acid, and butyric acid),^67^ with the latter possibly exerting beneficial effects such as appetite and glycemic control, anti-inflammation, immune regulation, and anti-tumor activity. With a few references related to XOS, how XOS combat tumor with the help of gut microbiota can be comprehensively described. To better understand the relationship between oligosaccharides and gut microbiota, XOS were selected as the primary research object to explore the interaction between XOS and gut microbiota. And hopefully, this review can facilitate researches of anti-tumor activity of other oligosaccharides with XOS as an example.

### 3.1 Condition to produce short chain fatty acids (SCFAs)

**Fig. 1:**
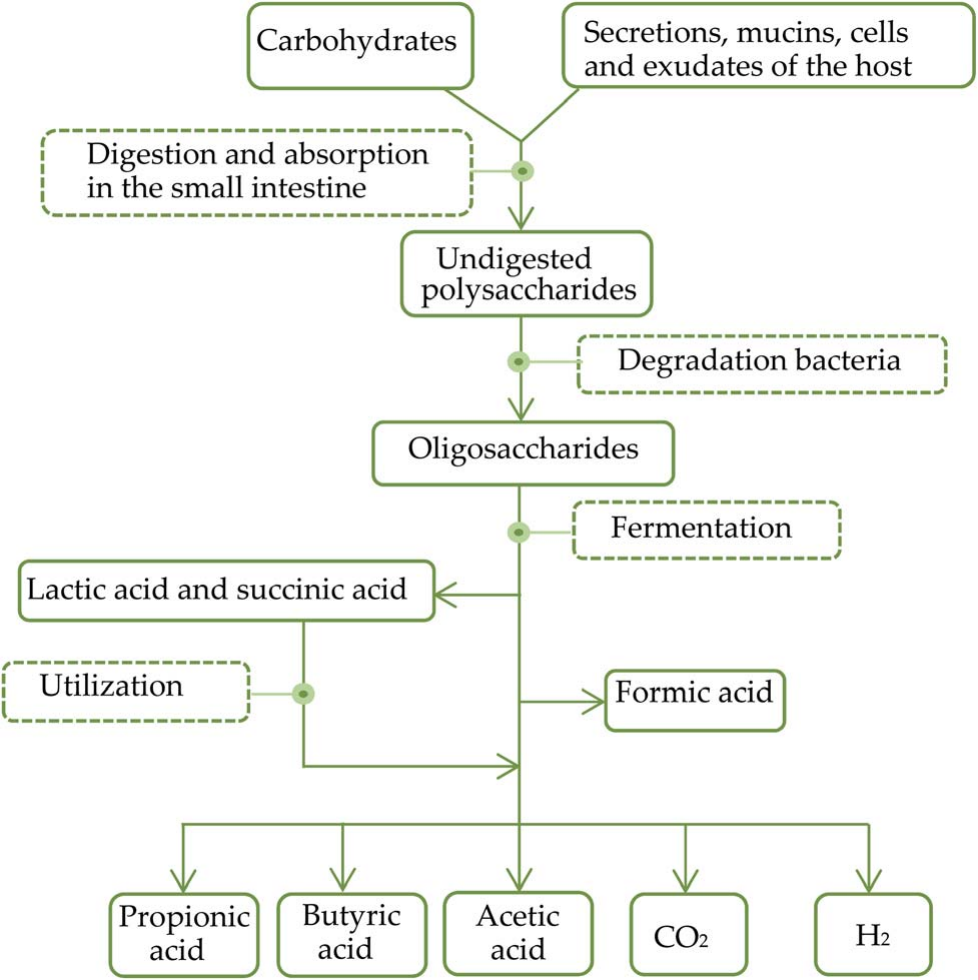
General metabolic pathways of carbohydrates in the intestinal tract.

SCFAs are organic carboxylic acids containing 1–6 carbon atoms. 90% of SCFAs are produced by anaerobic bacteria in the colon through fermentation of non-digested carbohydrates, such as oligosaccharides, non-starch polysaccharides and resistant starches. And the rest 10% are produced by dietary intake as well as protein metabolism. The general metabolic pathways in the intestinal tract are shown in Fig. 1. Healthy human intestines can produce about 50–100 mmol SCFAs per day.^68^ The common contents of intestinal SCFAs are butyric acid, acetic acid and propionic acid with a molar ratio of about 60 : 20 : 20.^69^ The typical bacteria that produced SCFAs include *Bacteroides*, *Clostridium*, *Bifidobacteria*, *Eubacterium*, *Streptococcus*, and *Streptococcus digestibilis*. The types and quantities of SCFAs produced by fermentation are various and play different roles in the intestinal tract due to the influence of fermentation substrates, bacteria and other factors.^70^ Most of SCFAs in the intestinal tract existing in the form of ions are mainly absorbed and utilized by transporters. Specific transporters of SCFAs, mainly distributing in colon cells and a small part in small intestine cells, include carboxylic acid transporters (McT-1) and sodium-coupled carboxylic acid transporters (smct-1).

The diversity of intestinal microbial genes provides a variety of digestive enzymes and biochemical pathways that are significantly different from those of the host. As some of the polysaccharides, such as AXs, have been reported to be possessed by a starch utilization system (Sus)-like system encoded by polysaccharide utilization loci (PUL) of the Bacteroidetes. Polysaccharides are hydrolyzed into oligosaccharides and transported into cell periplasm *via* a set of polysaccharide binding-proteins (SusD homologue; SusD_H_), glycolytic enzymes and TonB-dependent transporters (SusC homologue; SusC_H_).^71^ Oligosaccharides are further degraded into monosaccharides, SCFAs, CO_2_ and H_2_ in the periplasm. Meanwhile, when the sources of polysaccharides in food are to reduce, the utilization of endogenous polysaccharides in the mucus of the host digestive system can effectively compensate the energy needs of the host, promote the absorption of monosaccharides and SCFAs and the synthesis of triacylglycerols and participate in host energy metabolism. After colonization in a sterile intestinal tract, Bacteroidetes can stimulate the expression of sodium/glucose transporters (SGLT1) in the intestinal epithelium, and enhance the ability to absorb and utilize monosaccharides in the digestive system of the host.^72^

**Fig. 2:**
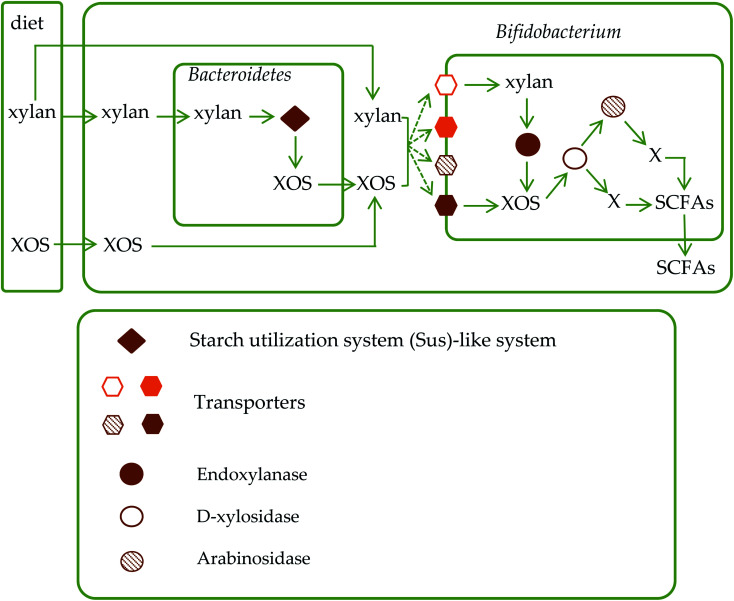
The metabolic pathways of xylan and XOS in the intestinal tract.

After entering the intestinal tract, xylan is degraded into XOS by glycoside hydrolases secreted by Bacteroidetes. XOS can be transported into the cell periplasm.^73^ The depolymerization pathway of XOS in cell periplasm of Bacteroidetes is unclear. Downstream metabolism of xylan and Bacteroidetes can be transported into *Bifidobacterium* or other bacteria through transporters and be depolymerized by carbohydrate-active enzymes which encoded by Gram positive PULs.^73^ XOS can be hydrolyzed into monosaccharides by d-xylosidase and arabinosidase secreted by gut microbiota,^74^ and then utilized to produce SCFAs such as acetic acid, propionic acid, butyric acid, lactic acid and succinic acid. The metabolic pathways are shown in Fig. 2. Geraylou *et al.*^75^ found that XOS can significantly increase the content of organic acids. Sarma *et al.*^76^ also found that the concentration of acetic acid increased significantly after the ingestion of arabinose–xylose-oligosaccharide.^77^ SCFAs, which, as organic acids, can lower the pH in the intestine, promote gastrointestinal motility, and inhibit the growth and reproduction of nitrate-reducing bacteria, and thus prevent the occurrence of intestinal diseases.^78^ SCFAs can affect immune function mechanisms of the host by regulating epidermal barrier, causing natural immunity to mediate inflammation, and inhibiting intestinal pathogen infection. And SCFAs inhibit the growth and proliferation of colorectal tumor cells, induce tumor cell differentiation and apoptosis, and play an anti-tumor role.^79,80^ However, there were opposite experimental results. For example, Soleimani *et al.*^81^ found that there was no significant difference between the concentrations of acetic acid, propionic acid and lactic acid in the feces of the experimental group and the control group after the intake of XOS. In these cases, it can be speculated that SCFAs produced by XOS are absorbed into the blood by the intestinal mucosa, leading to rapid changes of pH and the concentration of SCFAs in feces.^81^

### 3.2 XOS structure and microbiota fermentation

Different structures of XOS have different effects on the performance of gut microbiota. Studies were carried out from the aspects of polymerization degree (DP), substitution degree (DS), modification methods and substitution position of XOS. Different structures of XOS components were used for the study on the same bacteria *in vitro*, so their roles in the performance of beneficial bacteria could be understood.

It has been found that the biological activities of XOS mainly came from the components with low average DP. For example, the effect of xylotriose on *Bifidobacterium* proliferation *in vitro* is higher than that of xylopentaose, xylohexaose and mixed XOS, which shows that the DP of XOS will affect the performance of gut microbiota.^82^ The effect of the DS of XOS on gut microbiota can be reflected in its proliferation and fermentation rate. It was found that when xylobiose is replaced by arabinose at the O-3 position of the end xylose, bacterial growth on the substituted substrate decreases. This finding suggests that bacteria prefer the unsubstituted disaccharide to the substituted disaccharide.^22^ However, if the substituted oligosaccharides are cleaved into shorter oligosaccharides, the metabolic efficiency will be the same as that of the unsubstituted oligosaccharides. Different modification methods or substituents also affect the performance and metabolism of gut microbiota. Researchers have compared gut microbiota fermentation on the unsubstituted XOS with that on XOS substituted by arabinose (AXOS), acetyl (AcXOS) or glucuronic acid groups (GlcAmeXOS).^82^ For all fermentations described a differences could be made between the first stage of the fermentation (0–40 h) and a second stage (>40 h). In the first stage of the fermentations the pH decreased, whereas in the second stage the pH remained constant or even increased slightly. It is indicated that the fermentation rates of unsubstituted XOS and AXOS are faster than that of AcXOS or GlcAmeXOS in the first stage. In the first stage of the XOS and AXOS fermentations, acetate and lactate were mainly formed. Lactic acid bacteria (*e.g.*, *Lactobacillus* and *Enterococcus* species) and *Bifidobacterium* spp. may play an important role in this part of the fermentation, as they do not produce butyrate or propionate but they do produce acetate and lactate.^83^ And they can survive in the low pH environment. A high concentration of acids formed might be desirable because of a decrease in pH. And the growth of potentially pathogenic microorganisms and the growth of putrefactive bacteria will be inhibited.^83,84^ The preference for Bifidobacterial to ferment low-substituted XOS, both *in vitro* and *in vivo*, has been described previously.^85^ The latter observations corresponded well with our results that in the first stage of the fermentation of AcXOS and GlcAmeXOS in addition to acetate and lactate also propionate and some butyrate were formed. This is most likely due to the growth of several intestinal bacteria and not specifically of lactic acid bacteria. However, after an adaptation time, the GlcAmeXOS were fermented at the same rate as the XOS and AXOS.

So far, there has been no report on the similarities and differences between the XOS with different structures in the biological functions and their metabolic mechanisms mediated by gut microbiota. XOS with different structure make an unclear contribution to the performance of the gut microbiota, the types and composition of the metabolites during the metabolic process. Therefore, research in this area can fill the gap in the study of biological functions of XOS.

### 3.3 The change of gut microbiota

The interaction between XOS and gut microbiota occurs in the intestinal tract. After entering the intestinal tract, XOS can to be utilized by beneficial bacteria that produce acids such as Bifidobacteria and *Lactobacilli*. Beneficial bacteria such as *Bifidobacterium* can use XOS as nutrient substrates to proliferate in large numbers, thus improving the proportion of beneficial microbiota while reducing the ratio of harmful bacteria to improve the structure of gut microbiota.^86^ A large number of pure culture studies on Bifidobacteria have confirmed that XOS can be utilized by various Bifidobacteria (*B. bifidum*, *B. longum*, *B. catenulatum*, *B. lactis* and *B. adolescentis*).^87,88^ The studies on the colon model have reached the same conclusion.^89^ In addition, experiments in rats have also shown that XOS not only have a highly selective proliferation effect on Bifidobacteria in the intestinal tract but also prolongs its survival time.^90^

While selectively proliferating beneficial bacteria, XOS can also effectively inhibit the growth and reproduction of harmful bacteria. Moura^87^ found that XOS can be efficiently utilized by a variety of *Bifidobacterium*, *Lactobacillus* and *Bacillus etc.*, but can't be degraded by harmful bacteria such as *Escherichia coli*, *Enterococcus* and *Clostridium perfringens*. The mechanism may be that *Bifidobacterium* and other beneficial bacteria can secrete arabinase and d-xylosidase to hydrolyze XOS into monosaccharides, while harmful bacteria can't produce these enzymes.^91,92^ In addition, the structural characteristics of XOS themselves can also inhibit the growth of harmful bacteria.^22,93,94^

## 4 Anti-tumor activity of XOS

XOS have attracted the interest of researchers for their activity to combat tumor and prevent cancer. Although there is no detailed description of the specific process and mechanism of XOS exerting anti-tumor activity and cancer prevention, it is generally believed that XOS can selectively proliferate beneficial bacteria after entering the intestinal tract and being degraded by beneficial bacteria to produce active substances.

### 4.1 Anti-tumor activity of SCFAs

A large number of researches on the gut microbiome's role in infection and inflammation control have initially revealed how specific microbiota and their metabolites affect the natural immune system.^66^ SCFAs are mainly bacterial metabolites, playing wide roles in immunity.^95,96^ SCFAs enter cells through passive diffusion, vector-mediated transport, and interaction with GPCRs (g-protein-coupled receptors) in colon.^97^ SCFAs can affect immune function mechanisms of the host with various methods, including regulating epidermal barrier, causing natural immunity to mediate inflammation, and inhibiting intestinal pathogen infection. SCFAs can enhance the protective effect of the intestinal epidermis. The barrier layer outside the intestinal epidermis consists of mucus produced by AMPs (antimicrobial peptides) and specific intestinal epidermal cells (paneth cells and goblet cells). Recently, studies have shown that SCFAs can enhance the barrier function of these components, thus preventing diseases. In addition, SCFAs can induce an immune response in the host. SCFAs,^98^ for example, exert an anti-inflammatory effect on colonic macrophages and DCs by inhibiting the HDAC (histone deacetylase). Meanwhile, the production of SCFAs lowers the pH of the intestinal environment, thus inhibiting the proliferation of pathogenic bacteria and innate intestinal spoilage organisms and reducing the generation of endogenous carcinogens.^97^ SCFAs can also reduce the redox potential and have negative impacts on the redox reactions of coenzymes, which are essential to the growth and metabolism of harmful intestinal bacteria. Meanwhile, SCFAs can also stimulate intestinal peristalsis and shorten the time of chyme staying in the intestine, thus reducing the toxicity that harmful substances may cause to the host.

XOS are fermented by gut microbiota to produce SCFAs. Such SCFAs mainly include butyric acid, acetic acid and propionic acid, which all play roles in biological functions like anti-tumor and cancer prevention.^99^ In cell experiments, research found that physiological doses of acetic acid, propionic acid and butyric acid could inhibit the growth and proliferation of colorectal tumor cells, induce tumor cell differentiation and apoptosis, and play an anti-tumor role.^79^ Butyric acid plays a particularly important role in anti-tumor activity.^100^ Butyric acid induces the expression of β defensins through the muc2-dependent pathway. Killing harmful bacteria by inducing the expression of β defensins is one of the manifestations of the anti-tumor activity of butyric acid.^97^ In addition to be an important source of energy for colonic epithelial cells, butyric acid shows many properties in different cells, such as inhibiting cell growth in the early stages of the digestive tract, inducing differentiation and stimulating cytoskeleton formation. Butyric acid can slower or inhibit the growth of cells in many cell lines, such as liver cancer cells, colon cancer cells and pancreatic cancer cells. Butyric acid can also change the expression of genes related to cell differentiation.^101^ Butyric acid can also exert an anti-tumor effect by inhibiting histone deacetylase and telomerase activity.^102^ In addition, it has been found that the expression of butyric acid transporters MCT1 and SMCT1 on the colon cancer cell membranes decreased obviously, suggesting that the decline of transit and bioavailability of butyric acid may play a role in colon cancer development.^103,104^ Making efforts to supplement butyric acid or to enhance its transport effect may play a supporting role in the treatment of colon cancer.

Although the anti-tumor effect of SCFAs, especially butyric acid, can be affirmed, it has not been put into use in clinical practice. If it can be further studied to develop its clinical application, it will provide new ideas for the treatment of colon cancer and other diseases.

### 4.2 Anti-tumor activity of gut microbiota

XOS have proper prevention and treatment effects on malignant tumors such as colorectal cancer.^105^ It is indicated that XOS can increase the immune function of the host through the actions of beneficial bacteria such as *Bifidobacterium*.^106^ The surface molecular active substances of *Bifidobacterium* can induce tumor cell apoptosis by regulating the expression of related genes. For example, *Bifidobacterium adolescentis in vivo* can prevent colorectal cancer by inducing tumor cell apoptosis.^43^ Bifidobacteria also has immune activation, which can enhance the phagocytic activity of macrophages and directly kill tumor cells.^107^ Beneficial bacteria can secrete a variety of antibacterial substances to inhibit the growth and reproduction of the harmful intestinal bacteria, such as non-specific fatty acids and peroxides and high specificity bacteriocins.^108^ Bacteriocin is an antibacterial substance produced by *Bifidobacterium*, which can effectively inhibit harmful bacteria such as *Shigella*, *Salmonella*, and *E. coli*. *Bifidobacterium* can also secrete bile acid hydrolase, which converts the bile acid from a combined state to a free state to exert stronger antibacterial activity.

*Bifidobacterium* can not only obviously inhibit the development of tumor but also prevent the occurrence and development of a variety of tumors.^109^ Bifidobacteria can also acidify the intestinal environment, accelerate the excretion of tumor-causing substances, and shorten the contact time between tumor-causing substances and intestinal mucosa to reduce the occurrence of tumors. *Bifidobacterium* can increase the number of peripheral leukocyte cells and NK cells. As an immune adjuvant, *Bifidobacterium* can identify PP lymph nodes, activate intestinal lymph nodes, induce lymphocyte outflow through lymphatic vessels, and activate the immune system by lymphatic circulation.^110^ The role of Bifidobacteria in promoting immune function is also related to the secretion of sIg A.^111^ Bifidobacteria can enhance the activity of immune cells, and promote the production of intestinal Ig A plasma cells, thereby killing bacteria and viruses that invade the host and preventing the occurrence and deterioration of the disease.

Beneficial bacteria have prominent anti-tumor activity and cancer prevention activity. However, they have not been put into practical application due to the limitations of current research. Therefore, it is of great practical significance to further explore the mechanism of the anti-tumor effect of gut microbiota and develop mature application plans.

### 4.3 Anti-tumor activity of gut microbiota

Lignins are major class of natural products presented in the natural, and are formed through phenolic oxidative coupling processes in the plant.^112^ Lignins are formed by the dehydrogenative polymerization of three monolignols: *p*-coumaryl, *p*-coniferyl and sinapyl alcohols.^113^ Some polysaccharides in the cell walls of lignified plants are linked to lignin to form LCCs.^112^ Studies have shown that LCCs may be involved in the induction of anti-tumor, anti-microbial, anti-HIV and antioxidation activity.^112,114,115^ Polysaccharide portions of LCCs are composed of various types of sugars, such as glucose, arabinose, mannose, galactose, fucose and xylan.^114^ LCCs from bald cypress, birch and rice straw show an extremely broad molecular weight distribution from 1.5 to 85 kDa.^116^ The sweet taste of XOS makes it an ideal food additive. XOS prebiotics are a value-added product from lignocellulosic biorefineries based on autohydrolysis or related pretreatment technologies.^117^ When using XOS as a food additive, the residual lignin in the XOS can also alter the human intestinal tract and play an anti-tumor role.

The anti-tumor activity of LCCs is manifested in two aspects: *in vitro* cytotoxicity and endogenous TNF production. Generally, LCCs showed much lower cytotoxicity, compared with phenylpropenoid monomers,^118^ tannins^119^ and flavonoids.^120^ Natural lignified materials (250 μg mL^−1^) induced DNA fragmentation in human promyelocytic leukemia cells HL-60, whereas commercially available alkali-lignin and lignin sulfonate were inactive.^114^ After the mice were treated with LCCs, high level of endogenous TNF was induced, accompanied by hepatic accumulation of Kupffer cells. Endogenously produced TNF has been reported to induce anti-tumor activity and resistance against microbial infection.^121^ The endogenous TNF production primed by LCC decreased during the aging of mice and upon the tumor implantation into the mice,^122^ which suggests that the resistance of the hosts against microbial infection may decline with aging. Whether LCCs play an anti-tumor role mediated by gut microbiota is still unclear, and it should be further studied.

## 5 Conclusion

In this paper, the biological activities and structural characteristics of eight natural oligosaccharides were summarized. Taking XOS as an example, the relationship between XOS and gut microbiota was studied, and the process of anti-tumor activity mediated by gut microbiota was reviewed.

It has been suggested that there is a close relationship between XOS and gut microbiota. On the one hand, XOS can selectively proliferate beneficial bacteria while inhibiting the growth of harmful bacteria. On the other hand, XOS have influences on the fermentation features of gut microbiota, such as the fermentation rate or fermentation products. XOS can proliferate probiotics and stimulate probiotics to produce SCFAs. Then, through the effects of SCFAs and probiotics themselves, biological activities such as anti-tumor activity can be achieved. Therefore, by studying the changes and metabolic mechanism of gut microbiota and their fermentation products after adding oligosaccharides to the diet, we can better understand how oligosaccharides exert biological functions.

Future researches should further study the anti-tumor activity of XOS and other oligosaccharides mediated by gut microbiota. And the future researches can provide new ideas for the treatment of tumor diseases and for the development of new high-quality functional foods.

## Author contributions

R.-M. H. and J. L. conceived the project. Y.-L. W. wrote the manuscript. All authors proofread and edited the manuscript.

## Conflicts of interest

The authors declare no conflict of interest.

## Supplementary Material
